# Therapeutic Landscapes in Colorectal Carcinoma

**DOI:** 10.3390/medicina59050821

**Published:** 2023-04-23

**Authors:** Antonio Mario Scanu, Maria Rosaria De Miglio

**Affiliations:** Department of Medicine, Surgery and Pharmacy, University of Sassari, 07100 Sassari, Italy; scanu@uniss.it

Colorectal cancer (CRC) is a disease of major public health and socioeconomic concern. According to GLOBOCAN 2020, in terms of incidence and mortality rates, CRC classifies third and second, respectively; in 2020, over 1.9 million novel CRC cases and 935.000 deaths were reported to occur, accounting to 1 in 10 cancer cases and deaths [1]. Recently, a decreased CRC incidence in high-incidence countries has been reported, which might be ascribed to population-level changes regarding healthier lifestyle choices, as well as to increased colonoscopy screening and an improvement in therapies [1]. However, more recurrent favorable prognosis affecting adults aged ≥50 years hides the increasing rates of early-onset CRC and age at diagnosis <50 years in various countries. In 2018, in fact, to alleviate the increasing burden of early-onset CRC, the recommended age for screening initiation for individuals at average risk has been lowered from 50 to 45 years by the American Cancer Society [2]. In October 2020, the US Preventive Services Task Force issued a recommendation statement (https://uspreventiveservicestaskforce.org/uspstf/recommendation/colorectal-cancer-screening, accessed on 18 May 2021).

Almost 20% of CRC patients show metastases at diagnosis, and metastatic CRC (mCRC) is most often a non-curable disease [3]. The five-year survival rate is 90% at stage I, severely decreasing to around 10% at stage IV [4]. Due to the high frequency of metastases and drug resistance, it is still one of the hard-to-treat cancers, despite all the advances in CRC biology knowledge and therapeutic improvements. Given that CRC is a heterogeneous and multifactorial disease with extremely different prognoses and responses to treatment, it is crucial to understand specific pathway abnormalities in order to improve diagnosis, prognosis, and therapeutic approaches. The ability to identify specific biomarkers and detect unequivocal molecular targets associated with early cancer signs will provide valuable support to achieve new targeted therapies and decrease CRC mortality rates. Even though different crucial genes and pathways have already been associated with CRC biology, the prognostic and predictive roles of many of these genomic alterations are unknown and have no influence on treatment decisions in metastatic patients. Recently, the identification of KRAS and NRAS gene mutations has been adopted as an extensively recognized molecular test in mCRC clinical treatment [5]. In order to identify patients with a more aggressive clinical outcome, BRAF V600E mutation has been approved as a prognostic biomarker [5]. Moreover, a relationship between MSI-high CRC and an effective response to the immune checkpoint blockade through anti-PD1 therapy has been proven in mCRC patients [6].

Accordingly, interpreting more effectively available data and further investigating molecular mechanisms triggering CRC pathogenesis are vital steps in order to achieve a higher level of prevention, outcome, and therapy in patients affected by CRC.

This Special Issue, entitled “Therapeutic Landscapes in Colorectal Carcinoma”, provides us with 11 really opportune articles, 9 original articles and 2 reviews, which may provide us with a deeper insight into the latest advances involving knowledge on CRC from scientific, translational, and clinical points of view. Through this Special Issue, our main purpose was to shed light on some state-of-the-art research on CRC. Here, the reader will find papers on prognostic factors, as well as on responses to neoadjuvant and adjuvant CRC treatments. Finally, we discuss the research trends and hotspots for CRC therapies that emerged during the COVID-19 pandemic, such as the potential achievement of a transition from CRC clinical research to precision medicine, together with a special emphasis given to new single-cell-based techniques.

The most important cause of death in CRC is disease progression due to metastasis and drug resistance. Therefore, the identification of unambiguous molecular biomarkers to predict disease aggressiveness and drug response is needed. Interestingly, it has been observed that a higher expression of RIPK2, which acts as a critical mediator necessary in different immune and inflammatory pathways, was associated with a high expression of VEGFA and increased mortality in CRC patients, suggesting its promising role as a prognostic tool [7].

Neoadjuvant chemotherapy (NAC) for locally advanced CRC has become progressively more commonly administered in clinical settings; however, its applicability is currently under debate. Zeng et al. demonstrated that a combination of NAC and adjuvant chemotherapy was not only safe, but also caused a noteworthy reduction in the primary tumor size and stage; in addition, in a lower percentage of patients, a complete pathological response (pT0) was observed. Eventually, long-term outcomes were similar to results in patients who after diagnosis directly underwent surgery.

CRC can be distinguished as mismatch repair-deficient (dMMR), with high levels of microsatellite instability (MSI-H), mismatch repair proficiency (pMMR), and microsatellite stability (MSS). Approximately 15–30% of CRC patients are affected by MSI-H [8], which has emerged as an important predictor of sensitivity to immune checkpoint inhibitors (ICIs). Considering that the European Medicines Agency (EMA) has recently approved pembrolizumab as the first ICI in the treatment of dMMR–MSI-H mCRC, Lungulescu et al. assessed the regional variability of MSI-H CRC in Romania, observing a higher regional MSI-H prevalence (21%) compared to the literature; they suggested that analyzing both geographical variations and clinical features in CRC patients is essential as advanced therapies, diagnostic tools, and innovative methods of treatments delivery are regularly being developed [9].

For instance, the study of patients’ clinical course after the diagnosis of de novo CRC after liver transplantation, with an emphasis on the influence of immunosuppressive management, showed a significantly enhanced survival rate when the immunosuppressive therapy was reduced in an individualized manner, leading to an optimal oncological therapy and higher survival rates [10].

Chen et al. conducted a nationwide, large-scale, retrospective cohort study to compare the effectiveness of adjuvant chemotherapy based on uracil and tegafur (UFT) in patients with stage II CRC; the study showed that, in the 15-year follow-up cohorts, UFT did not induce differences compared to the observation group in terms of disease-free survival (DFS) and overall survival (OS) rates. However, DFS notably increased in patients with stage IIA CRC treated with UFT as postoperative adjuvant chemotherapy compared with DFS in the observation group [11].

Seeing the scarce responses to standard systemic treatment in BRAF-mutated mCRC patients, through a single-center case series that included patients with BRAF-mutated mCRC in Asia, Yeh et al. analyzed the real effects of triplet therapy (dabrafenib, trametinib, and panitumumab) after previous systemic treatment failure. An adequate safety profile and acceptable treatment efficacy resulted from the study. Moreover, an interestingly higher OS was discovered in patients with left-sided mCRC than in patients with right-sided tumor [12].

Considering that the response to BRAF inhibitors of BRAF-mutated mCRC is rather brief, and progression is the rule, through an in silico study, Voutsadakis et al. suggested that targeted therapies for CRC showing BRAF mutations with or without PIK3CA mutations can be improved on the basis of the global molecular environment of these disease. The results showed that CRCs with BRAF mutations, and with or without PIK3CA mutations, vary in their MSI status and mimic CRC tissues with APC and TP53 mutations. CTNNB1, WRN, and CAD affected OS. Additionally, BRAF inhibitor sensitivity in CRC cell lines is shown by SACS mutations and PRKN loss [13].

Patients with mCRC show a poor prognosis despite the therapeutic options currently available. Regorafenib is an oral tyrosine kinase inhibitor that can be administered to treat refractory mCRC; however, no effective predictive markers for regorafenib treatment have been identified yet. De Summa et al. assessed somatic mutations of genes involved in immunological and inflammatory responses using an NGS platform to identify potential biomarkers in mCRC patients long and short responded to regorafenib. These results underline the presence of mutations in TGFBR1, TGFBR2, and TGFBR3 genes, suggesting the role of the TGF-β pattern in a prolonged response to the drug [14].

Voutsadakis et al. analyzed the therapeutic implications of 20q11.21 amplification in 12 CRC cell lines. Amplified 20q11.21 cell lines are sensible to different tyrosine kinase inhibitors and no-responders to drugs targeting the mitotic apparatus and microtubules. CRISPR and RNAi dependency analysis identified YAP1 and JUP as recurrent gene dependencies in cell lines. Therefore, amplified 20q11.21 gene cell line models of CRC with resistance or sensitivity to various drug categories could be adopted within in vitro models to favor clinical drug development in this tumor [15].

CRC shows heterogeneous genomic, epigenomic, and transcriptomic aberrations. Intra-tumoral heterogeneity (ITH) can be observed within a tumor in which cancer cell subpopulations with diverse genomic characters exist in a patient. As reported by Angius et al., ITH analysis is a promising new frontier that lays the basis toward effective CRC diagnosis and treatment. Genome and transcriptome sequencing, together with editing technologies, are transforming biomedical research, and represent the most encouraging tools for defeating unmet clinical and research challenges. Bulk and single-cell next-generation sequencing are recognizing genomic and transcriptional heterogeneity in primary and metastatic tumors [16].

Finally, Kopel et al. discussed research trends and hotspots for CRC management in the period of COVID-19 pandemic emergency. The authors suggested that the COVID-19 pandemic caused the world to pause and adopt lockdown measures that block CRC screening programs, which resulted in a dramatic increase in late-stage CRC cases and a general loss of life years due to the lack of appropriate treatments for CRC patients [17].

This Special Issue, titled “Therapeutic Landscapes in Colorectal Carcinoma”, illustrates a stimulating collection of articles written by international specialists that can promote discussions and ideas among colleagues working on CRC. We hope that it can encourage translational and interdisciplinary collaborations, leading to a definitive understanding of strategies to overcome and inhibit CRC progression and metastasis.
